# Black-white racial disparities in sepsis: a prospective analysis of the REasons for Geographic And Racial Differences in Stroke (REGARDS) cohort

**DOI:** 10.1186/s13054-015-0992-8

**Published:** 2015-07-10

**Authors:** Justin Xavier Moore, John P. Donnelly, Russell Griffin, Monika M. Safford, George Howard, John Baddley, Henry E. Wang

**Affiliations:** Department of Emergency Medicine, University of Alabama School of Medicine, 619 19th Street South, OHB 251, Birmingham, AL 35249 USA; Department of Epidemiology, University of Alabama at Birmingham, Birmingham, Alabama USA; Division of Preventive Medicine, Department of Medicine, University of Alabama School of Medicine, Birmingham, Alabama USA; Division of Infectious Diseases, Department of Medicine, University of Alabama School of Medicine, Birmingham, Alabama USA; Department of Biostatistics, University of Alabama at Birmingham, Birmingham, Alabama USA

## Abstract

**Introduction:**

Sepsis is a major public health problem. Prior studies using hospital-based data describe higher rates of sepsis among black than whites participants. We sought to characterize racial differences in incident sepsis in a large cohort of adult community-dwelling adults.

**Methods:**

We analyzed data on 29,690 participants from the Reasons for Geographic and Racial Differences in Stroke (REGARDS) cohort. We determined the associations between race and first-infection and first-sepsis events, adjusted for participant sociodemographics, health behaviors, chronic medical conditions and biomarkers. We also determined the association between race and first-sepsis events limited to first-infection events. We contrasted participant characteristics and hospital course between black and white sepsis hospitalizations.

**Results:**

Among eligible REGARDS participants there were 12,216 (41.1 %) black and 17,474 (58.9 %) white participants. There were 2,600 first-infection events; the incidence of first-infection events was lower for black participants than for white participants (12.10 vs. 15.76 per 1,000 person-years; adjusted HR 0.65; 95 % CI, 0.59-0.71). There were 1,526 first-sepsis events; the incidence of first-sepsis events was lower for black participants than for white participants (6.93 vs. 9.10 per 1,000 person-years, adjusted HR 0.64; 95 % CI, 0.57-0.72). When limited to first-infection events, the odds of sepsis were similar between black and white participants (adjusted OR 1.01; 95 % CI, 0.84-1.21). Among first-sepsis events, black participants were more likely to be diagnosed with severe sepsis (76.9 % vs. 71.5 %).

**Conclusion:**

In the REGARDS cohort, black participants were less likely than white participants to experience infection and sepsis events. Further efforts should focus on elucidating the underlying reasons for these observations, which are in contrast to existing literature.

## Introduction

Sepsis is a clinical disease characterized by a systemic inflammatory response to a severe infection. Sepsis is an important public health problem, responsible for more than 750,000 hospitalizations, 570,000 Emergency Department visits and 215,000 deaths annually [1]. In the United States health system, sepsis care exceeds $16.7 billion annually [1]. Sepsis survivors are at increased risk of long-term death, cognitive impairment and functional limitation [1–9].

Population-based studies have shown that black individuals have higher rates of sepsis, hospitalization mortality, and are twice more likely to develop sepsis than white individuals [3–6, 10–13]. Over a 22-year study period black participants had higher rates of sepsis than white participants [5]. Mayr et al. found that higher incidence of sepsis among black patients was attributed to higher infection-related hospitalization rates and a higher risk of acute organ dysfunction [6]. Barnato, et al. found that even while adjusting for poverty, black patients still experienced a higher population-based incidence of severe sepsis [3]. An important feature of these studies was their use of hospital discharge data for identifying sepsis events without information on initial clinical presentation or comorbid conditions. Furthermore, these studies did not distinguish hospital-acquired from community-acquired sepsis events, nor compare hospitalized sepsis patients with non-hospitalized individuals.

The REasons for Geographic and Racial Differences in Stroke (REGARDS) cohort is one of the largest longitudinal cohorts of community-dwelling adults in the United States [14]. The objective of this study was to characterize racial differences in sepsis incidence in the REGARDS cohort.

## Methods

### Ethics and consent statement

The Institutional Review Board of the University of Alabama at Birmingham approved this study. We obtained informed consent from all participants of the study during baseline visit, and we also obtained consent for subsequent blood samples.

### Study design and data source

We performed a prospective cohort analysis using REGARDS, one of the largest ongoing national longitudinal cohorts of community-dwelling adults in the United States [14]. Designed to evaluate geographic and black-white differences in risk factors for stroke, REGARDS recruited 30,239 black and white participants aged ≥45 years. The cohort is 45 % male and 41 % African American, and 69 % of participants are >60 years old. REGARDS recruited participants between January 2003 and October 2007. In 6-month intervals, REGARDS contacted the participants by telephone to identify any hospitalizations experienced by the participant. REGARDS personnel retrieved medical records for specific health events. REGARDS reviewed death certificates, medical records, and interviewed proxies to discern whether a participant died and the causes of the death. Further details of REGARDS study methods are described elsewhere [14].

While the objective of REGARDS was to identify and characterize stroke events, the population of REGARDS included community-dwelling adults at healthy baseline - not just individuals who had suffered a prior stroke. The REGARDS-sepsis ancillary study used the infrastructure of the parent REGARDS study to independently identify sepsis hospitalizations.

### Identification of serious infection and sepsis events

We included all hospitalization events reported over a 10-year follow-up period ending December 31, 2012. The primary outcomes of this study were 1) first hospitalization for a serious infection, and 2) hospitalization for sepsis. Using the taxonomy of Angus et al., we identified all hospitalizations (Emergency Department visits and/or hospital admission) attributed by participants to a serious infection [1]. Two trained reviewers evaluated information from the corresponding medical record, confirming the presence of a serious infection based upon diagnoses documented in the Emergency Department or admission physician record. Discordances were adjudicated among abstractors, with additional physician review as needed.

Using international consensus definitions, we defined sepsis events as hospital admission for a serious infection with the presence of at least two systemic inflammatory response syndrome (SIRS) criteria, including heart rate >90 beats/minute, fever (temperature >38.3 °C or <36 °C), tachypnea (>20 breaths/minute) or PCO2 <32 mmHg, and leukocytosis (white blood cells >12,000 or <4,000 cells/mm3 or >10 % band forms) [1]. We used vital signs and laboratory test results for the initial 28 hours of hospitalization. Because of our focus on community-acquired (vs. hospital-acquired) sepsis, we did not include sepsis developing at later points during hospitalization. We did not include organ dysfunction in the definition of sepsis. Initial review of 1,349 hospital records indicated excellent inter-rater agreement for presence of serious infection (kappa = 0.92) and the presence of sepsis (kappa = 0.90) upon hospital presentation.

### Participant characteristics

Participant demographics included self-reported age, race, sex, income, education, and geographic location. Health behaviors included tobacco and alcohol use. Smoking status included current, past and never. We defined alcohol use as moderate (one drink per day for women or two drinks per day for men) and heavy alcohol use (>1 drink per day for women and >2 drinks per day for men), per the National Institute on Alcohol Abuse and Alcoholism classification [15].

Baseline medical conditions included atrial fibrillation, chronic lung disease, coronary artery disease, deep vein thrombosis, diabetes, dyslipidemia, hypertension, myocardial infarction, obesity, peripheral artery disease, and stroke. We defined atrial fibrillation by participant self-report or baseline electrocardiogram (ECG) evidence of an atrial fibrillation event. Chronic lung disease was defined as participants with a history of prescribed pulmonary medication. Coronary artery disease was classified in participants with a history of heart disease (self-reported myocardial infarction (MI), coronary artery bypass graft, bypass, angioplasty, or stent) or ECG evidence. Diabetes was defined as a fasting glucose level ≥126 mg/L (or a glucose level ≥200 mg/L for those not fasting) or the use of insulin or oral hypoglycemic agents. Dyslipidemia consisted of low-density lipoprotein cholesterol >130 mg/dL, or use of lipid-lowering medications. Hypertension included systolic blood pressure ≥140 mm Hg, diastolic blood pressure ≥90 mm Hg, or the reported use of antihypertensive agents. MI in participants was defined by ECG evidence or self-reports of MI in the participant’s history. Obesity was defined as body mass index (BMI) ≥30kg/m^2^ in addition to gender-specific waist circumference [16].

REGARDS did not collect information on pulmonary conditions such as asthma and chronic obstructive pulmonary disease. Therefore, we defined participant use of pulmonary medications as a proxy for chronic lung disease. Obtained from each participant’s medication inventory, pulmonary medications included beta-2 adrenergic agonists, leukotriene inhibitors, inhaled corticosteroids, combination inhalers, and other pulmonary medications such as ipratropium, cromolyn, aminophylline and theophylline. Other medical conditions such as; deep vein thrombosis, peripheral artery disease, and stroke were based upon self-reports.

### Determination of biomarkers

Biomarkers examined in this study included serum high-sensitivity C-reactive protein (hsCRP), urinary albumin-to-creatinine ratio (ACR), Cystatin C (Cyst-C), and estimated glomerular filtration rate (eGFR). We estimated glomerular filtration (eGFR) rate using the Chronic Kidney Disease Epidemiology collaboration (CKD-EPI) equation [17]. We dichotomized all biomarkers as abnormal or normal within statistical models. We defined eGFR <60 ml/min/1.73 m^2^ as abnormal. Consistent with our prior study, we defined hsCRP >3.0 mg/dL as abnormal [18]. We defined Cyst-C measurements above the fourth quartile of values observed in the REGARDS cohort (≥1.1 mg/dL) as abnormal. We defined ACR ≥30 mg/g as abnormal. Further details of REGARDS biomarkers are defined elsewhere [18–20].

### Hospital course

We defined hospital course as information about selected events during the hospital stay. Hospital course variables included infection type, presence of severe sepsis, sequential organ failure assessment (SOFA) for respiratory, renal hepatic, cardiovascular, hematologic, and neurologic systems, mortality in emergency department sepsis (MEDS) score, ICU admission, hospital mortality, and long-term mortality. We defined severe sepsis as the presence of sepsis and organ dysfunction.

### Analysis

We compared racial differences in baseline demographics and hospital presentation characteristics using the Chi-square test for categorical characteristics, analysis of variance (ANOVA) for continuous characteristics (i.e., age), and the Kruskal-Wallis test for non-parametric continuous variables (i.e., biomarker levels). To estimate the relative rates of first infection and first sepsis in black and white participants, we fit a series of Cox proportional hazards models with time to first infection and time to first sepsis as endpoints. We censored individuals at the time of their event, death, or end of follow up (31 December 2012). We adjusted the estimates for participant sociodemographic data, health behaviors, chronic medical conditions and biomarkers. In a separate sensitivity analysis, we used the Fine and Gray model to examine all-cause mortality among all participants as a potential competing risk for sepsis events [21].

To determine the odds of sepsis among those experiencing an infection, we fit a multivariable logistic regression model assessing the association between sepsis and race, limiting the analysis to those with serious infection. We adjusted the model for sociodemographic data, health behaviors, chronic medical conditions, and biomarkers.

Due to the number of missing values for several variables (income 12.4 %, Cyst-C 6.8 %, CRP 6.4 %, albumin 4.7 %, creatinine 4.5 %), we performed the multivariable modeling with multiple imputation using chained equations (Stata MI suite), pooling regression coefficient estimates for each model across 10 imputations using Rubin’s rules [22, 23]. We used SAS version 9.4 and Stata version 13 for all analyses. We considered *p* values ≤0.05 statistically significant.

## Results

### Baseline characteristics

**Fig. 1:**
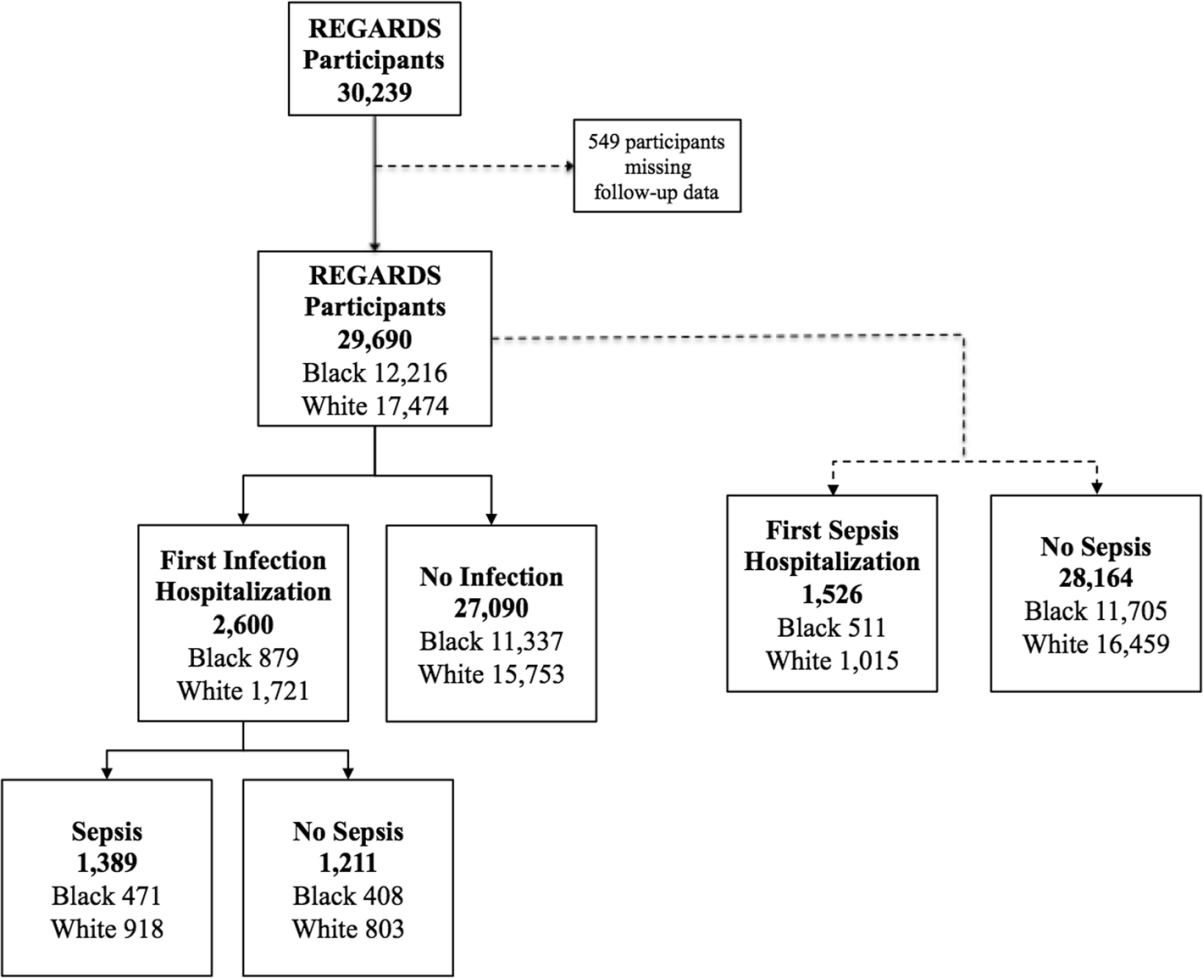
Flowchart of participants in the Reasons for geographic and racial differences in stroke (REGARDS) study who were included in the analysis

Among 30,239 available participants, 549 participants were excluded from analysis due to incomplete follow-up time, corresponding to 29,690 participants (Fig. 1). Among the 29,690 participants included in this analysis, there were 12,216 (41.1 %) black participants and 17,474 (58.9 %) white participants (Table 1). Black and white participants had a similar distribution of age, but black participants were more likely to be female than white participants. Black participants reported lower education and income. Tobacco use was similar between races, but black participants were less likely than white participants to use alcohol. Black participants were more likely to have a history of diabetes, hypertension, obesity and stroke. White participants were more likely to have a history of alcohol use, atrial fibrillation, chronic lung disease, coronary artery disease, dyslipidemia, and myocardial infarction.

**Table 1:** Baseline characteristics of participants in the Reasons for geographic and racial differences in stroke (REGARDS) study, stratified by race

	Black	White	*P* value*
	(n = 12,216)	(n = 17,474)	
Age^†^	64.2 (9.3)	65.6 (9.5)	<0.001
Education (%)			
≤ High school	2,429 (19.9)	1,279 (7.3)	<0.001
High school graduate	3,401 (27.9)	4,266 (24.4)	
Some college	3,237 (26.5)	4,713 (27.0)	
College grad or more	3,135 (25.7)	7,207 (41.3)	
Income (%)			
≤$20 000	3,257 (26.7)	2,086 (11.9)	<0.001
$20 000−$34 000	3,223 (26.4)	3,951 (22.6)	
$35 000−$74 000	3,100 (25.4)	5,708 (32.7)	
$75 000 and Above	1,088 (8.9)	3,609 (20.7)	
Refuse	1,548 (12.7)	2,120 (12.1)	
Geographic region (%)			
Stroke belt^a^	6,261 (51.3)	10,239 (58.6)	<0.001
Non-belt^b^	5,955 (48.8)	7,235 (41.4)	
Sex			
Male	4,622 (37.8)	8,713 (49.9)	<0.001
Female	7,594 (62.2)	8,761 (50.1)	
Tobacco use			
Never	5,521 (45.4)	7,861 (45.1)	<0.001
Past	4,529 (37.3)	7,381 (42.4)	
Current	2,107 (17.3)	2,177 (12.5)	
Alcohol Use			
None	8,610 (72.4)	9,632 (55.9)	<0.001
Moderate	3,002 (25.2)	6,689 (38.9)	
Heavy	288 (2.4)	889 (5.2)	
Baseline medical condition			
Atrial fibrillation	921 (7.8)	1,628 (9.5)	<0.001
Chronic lung disease	960 (7.9)	1,771 (10.1)	<0.001
Coronary artery disease	1,857 (15.5)	3,375 (19.6)	<0.001
Deep vein thrombosis	600 (4.9)	955 (5.5)	0.02
Diabetes	3,767 (31.0)	2,936 (16.9)	<0.001
Dyslipidemia	6,435 (55.3)	10,526 (62.1)	<0.001
Hypertension	8,706 (71.4)	8,840 (50.7)	<0.001
Myocardial infarction	1,396 (11.7)	2,323 (13.5)	<0.001
Obesity	7,684 (63.0)	8,181 (46.9)	<0.001
Peripheral artery disease	293 (2.4)	370 (2.1)	0.1
Stroke	982 (8.1)	914 (5.3)	<0.001
Abnormal biomarker levels^€♯^			
C-reactive protein (CRP)	2.9 (1.2–6.5)	1.9 (0.9 4.3)	<0.001
Albumin creatinine ratio (ACR)	8.1 (4.7–20.9)	7.2 (4.7–14.4)	<0.001
Cystatin C	0.9 (0.8–1.1)	1.0 (0.8–1.1)	<0.001
Estimated glomerular filtration rate (eGFR)	91.5 (73.9–106.8)	85.5 (72.2–95.0)	<0.001

^†^Mean (standard deviation). ^a^Defined as the states of Alabama, Arkansas, Georgia, Louisiana, Mississippi, North Carolina, South Carolina, and Tennessee; ^b^defined as all other states. *Significance determined using Chi-square test, analysis of variance, or the Kruskal-Wallis test. ^€^Median (IQR). ^♯^CRP in mg/dL; eGFR in ml/min/1.73 m^2^; ACR in mg/g; Cystatin C in mg/dL

### Rates of serious infection and sepsis

**Fig. 2:**
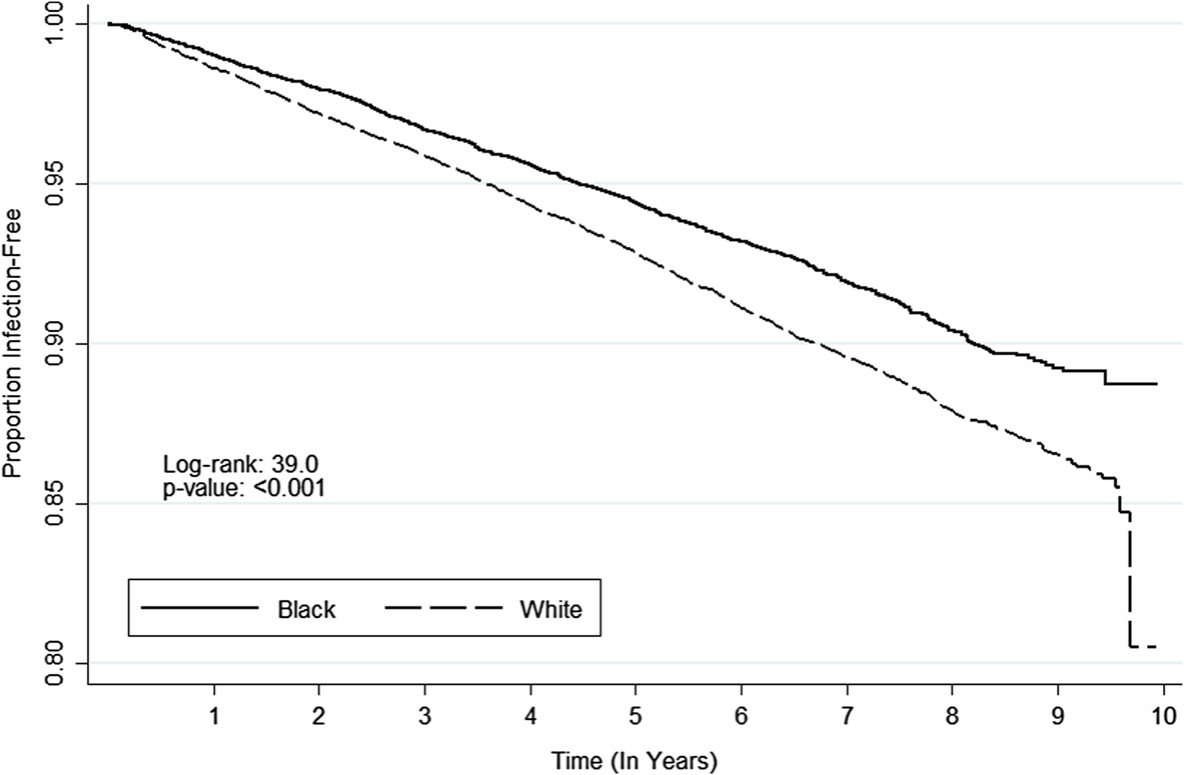
Kaplan-Meier plot for time to first-infection event, stratified by race

There were 2,600 first-infection events over the observation period. The incidence of first-infection events was lower for black participants than those who were white (12.10 vs. 15.76 per 1,000 person-years, hazard ratio (HR) 0.77; 95 % CI, 0.71, 0.84) (Fig. 2, Table 2). The observed reduced hazard of first infection among black participants was more pronounced after adjustment for demographic data, health behaviors, chronic medical conditions, and biomarkers (adjusted HR 0.65; 95 % CI, 0.59, 0.71) (Fig. 2, Table 2).

**Table 2:** Association between race and first-infection and first-sepsis events

	Black vs. white						
	Events, number (%)	Incidence density per 1,000 person-years	Crude hazard ratio*	Model 1^a^	Model 2^b^	Model 3^c^	Model 4^d^
		(95 % CI)	(95 % CI)	(95 % CI)	(95 % CI)	(95 % CI)	(95 % CI)
First-infection event							
White	1721 (5.8)	15.76 (15.65, 15.87)	0.77 (0.71, 0.84)	0.70 (0.64, 0.76)	0.68 (0.63, 0.75)	0.65 (0.59, 0.71)	0.65 (0.59, 0.71)
Black	879 (3.0)	12.10 (11.95, 12.26)					
First-sepsis event							
White	1015 (3.4)	9.10 (8.96, 9.25)	0.76 (0.69, 0.85)	0.68 (0.61, 0.76)	0.67 (0.60, 0.75)	0.63 (0.56, 0.71)	0.64 (0.57, 0.72)
Black	511 (1.7)	6.93 (6.73, 7.13)					
	Events, number (%)	-	Crude odds ratio‡	Model 1^a^	Model 2^b^	Model 3^c^	Model 4^d^
			(95 % CI)	(95 % CI)	(95 % CI)	(95 % CI)	(95 % CI)
Sepsis (given first-infection event)							
White	918 (53.4)	-	1.01 (0.86, 1.19)	0.99 (0.84, 1.18)	0.99 (0.83, 1.17)	1.00 (0.83, 1.19)	1.01 (0.84, 1.21)
Black	471 (53.5)	-					

^a^Adjusted for sex, age, and geographic region, education level, and income. ^b^Adjusted for model 1 covariates plus tobacco and alcohol use. ^c^Adjusted for model 2 covariates plus baseline chronic medical conditions. ^d^Adjusted for model 3 covariates plus biomarkers. *Estimated from Cox proportional hazard model. ^‡^Estimated from logistic regression

**Fig. 3:**
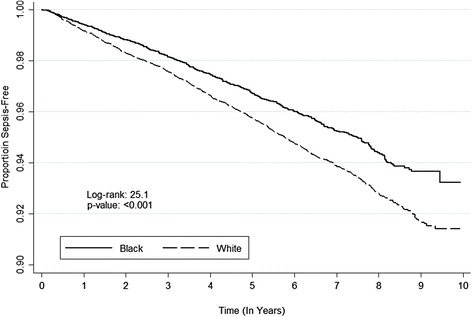
Kaplan-Meier plot for time to first sepsis, stratified by race

Among study participants there were 1,526 first-sepsis events over the observation period. The incidence of first-sepsis events was lower for black participants than white participants (6.93 vs. 9.10 per 1,000 person-years, HR 0.76; 95 % CI, 0.69, 0.85) (Fig. 3, Table 2). Similar to results for infection, the association was more pronounced after adjustment for demographics, health behaviors, chronic medical conditions, and biomarkers (adjusted HR 0.64; 95 % CI, 0.57, 0.72) (Fig. 3, Table 2).

Among the 2,600 first-infection events, 1,389 fulfilled sepsis criteria. Limited to the first-infection participants, the odds ratio (OR) for sepsis was similar between races (OR 1.01; 95 % CI, 0.86, 1.19). This association did not change after adjustment for confounders (adjusted OR 1.01; 95 % CI, 0.84, 1.21).

### Hospital presentation and course

Within the 2,600 first-infection events, infection types, severe sepsis frequency, SOFA scores, MEDS scores, ICU admission, hospital mortality, and long-term mortality were similar in black and white participants (Table 3). Among 1,526 first-sepsis events, black participants had higher proportions of severe sepsis and slightly higher SOFA scores (Table 4). Infection types, MEDS scores, ICU admission, hospital mortality and long-term mortality were similar in black and white participants.

**Table 3:** Hospital presentation and course for first-infection events stratified by race

	Black	White	*P* value*
	(n = 879)	(n = 1,721)	
Infection type (%)			0.06
Lung	364 (41.4)	705 (41.0)	
Kidney	168 (19.1)	311 (18.1)	
Abdominal	132 (15.0)	338 (19.7)	
Skin	117 (13.3)	214 (12.4)	
Sepsis	44 (5.0)	71 (4.1)	
Other	54 (6.1)	82 (4.8)	
Severe sepsis (%)	360 (41.0)	662 (38.5)	0.2
SOFA score**	2 (1-4)	2 (1-3)	0.06
MEDS score***	3 (3-6)	3 (3-9)	0.2
Admission to ICU (%)	65 (7.4)	133 (7.7)	0.8
Hospital mortality (%)	59 (6.7)	104 (6.0)	0.5
Long-term mortality (%)	227 (25.8)	404 (23.4)	0.2

*Estimated using either the Chi-square or Wilcoxon test. **Sequential organ failure assessment (*SOFA*) score, 28 hours, median (IQR). ***Mortality in Emergency Department sepsis (*MEDS*) score, median (IQR)

**Table 4:** Hospital presentation and course for first-sepsis events stratified by race

	Black	White	*P* value*
	(n = 511)	(n = 1,015)	
Infection type (%)			
Lung	235 (46.0)	503 (49.5)	0.04
Kidney	93 (18.2)	167 (16.4)	
Abdominal	66 (12.9)	166 (16.3)	
Skin	47 (9.2)	76 (7.5)	
Sepsis	42 (8.2)	61 (6.0)	
Other	28 (5.5)	44 (4.3)	
Severe sepsis (%)	393 (76.9)	726 (71.5)	0.02
SOFA score**	2 (1-4)	2 (1-3)	0.04
MEDS score***	3 (3-6)	3 (3-9)	0.2
Admission to ICU (%)	63 (12.3)	125 (12.3)	1.0
Hospital mortality (%)	53 (10.4)	87 (8.6)	0.3
Long-term mortality (%)	162 (31.7)	296 (29.2)	0.3

*Estimated using either the Chi-square or Wilcoxon test. **Sequential organ failure assessment (*SOFA*) score, 28 hours, median (IQR). ***Mortality in Emergency Department sepsis (*MEDS*) score, median (IQR)

### Sensitivity analysis

**Fig. 4:**
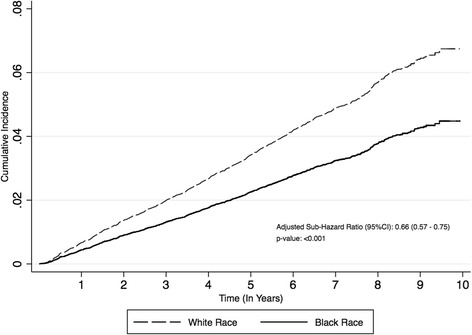
Competing Risks Analysis: Cumulative incidence function and adjusted sub-hazard ratio, stratified by race

When accounting for all-cause mortality as a competing risk, the increased rates of sepsis among white participants persisted (adjusted sub-HR 0.66; 95 % CI, 0.57, 0.75) (Fig. 4).

## Discussion

Prior epidemiological studies using hospital discharge data have reported higher incidence of sepsis and severe sepsis among black patients compared to those who were white (Table 5) [3–6, 10–13]. However, in this population-based study of community-dwelling adults in the REGARDS cohort, we found that black participants were at lower risk of developing sepsis than white participants. This contrasting racial disparity appeared to be due to lower rates of hospitalization for a serious infection. Once hospitalized for a serious infection, the odds of sepsis were similar in black and white participants. Among those hospitalized for sepsis, black participants were slightly more likely have severe sepsis, but other aspects of the hospital course were similar between races.

**Table 5:** Association between sepsis and race as determined in eight studies

Authors	Data	Method for sepsis case identification	Study Period	Population age (mean)*	Primary outcome	Sepsis rate ratio**
						black vs. white
Baine et al., 2001 [10]	US Medicare discharge data	ICD-9-CM discharge diagnoses	1991−1998	65−89 years	Septicemia	Black men RR 2.44
						Black women RR 2.13
Barnato et al., 2008 [3]	Six-state hospital discharge and US Census data	ICD-9-CM discharge diagnoses	2001	All ages (36.1)	Severe sepsis	Black RR 1.44;
						95 % CI 1.42, 1.46
Dombrovskiy et al., 2007 [4]	New Jersey state discharge data	ICD-9-CM discharge diagnoses	2002	≥18 years	Sepsis	Black RR 2.28
Esper et al., 2006 [11]	National representative sample of discharge data	ICD-9-CM discharge diagnoses	1979−2003	(60.5)	Sepsis	Mean annual black RR 1.90;
						95 % CI 1.82, 1.98
Martin et al., 2003 [5]	National representative sample of discharge data	ICD-9-CM discharge diagnoses	1979−2000	(60.8)	Sepsis	Mean annual black RR 1.90;
						95 % CI 1.81, 2.00
Mayr et al., 2010 [6]	Seven-state hospital discharge and US Census data.	ICD-9-CM discharge diagnoses	2005	All ages	Severe sepsis	Black IRR 1.67
McBean et al., 2001 [12]	US Medicare discharge data.	ICD-9-CM discharge diagnoses	1986−1997	≥65 years	Septicemia	Black RR 1.97
Richardus et al., 2001 [13]	National Longitudinal Mortality Study	ICD-9-CM discharge diagnoses	1979−1989	≥20 years	Septicemia	Black RR 1.87;
						95 % CI 1.35, 2.58

*Means are provided in parentheses - some studies did not report age ranges. **Confidence intervals were not provided by all studies (*p* values were used to show significance). *RR* relative risk IRR incidence rate ratio

The differences in observed race-sepsis associations between the current and prior studies could be explained by differences in the identification of sepsis events. Previous studies have applied ICD-9 taxonomies of Martin, et al. and Angus, et al. in order to identify sepsis and severe sepsis hospitalizations (Table 5) [3–6, 10–13]. This approach could lead to documentation bias, and research suggests that the use of discharge diagnoses tends to have poor sensitivity to detect sepsis [24, 26]. For example, a recent study concluded that ICD-9 discharge diagnoses were highly specific (94.6 %) but had poor sensitivity (27.6 %) for detecting community-acquired sepsis events. [26]. Due to the fact that present-on-admission flags are not commonly available in discharge datasets used for research purposes, prior sepsis studies were also unable to disentangle early community-acquired sepsis from later hospital-acquired sepsis. Moreover, documentation bias is plausible if physicians were more likely to record sepsis, infection and organ dysfunction diagnoses based on the patient’s race.

In contrast to prior work, our study employed comprehensive chart review in the definition of sepsis events. By using a definition that encompassed laboratory measurements and documentation of an infection, it is plausible that differences in sepsis detection could be attributable to hospital or ED practices. Specifically, patterns of initial diagnostic testing may have varied between black and white participants based on the site of presentation, leading to differing rates of sepsis. This assertion is supported by prior research, which indicates that black patients receive inferior care, such as lower quality and intensity of care [27]. Although we were not able to explore differences in hospital quality in the current analysis, this is an intriguing area of future research.

Studies performed across a wide range of disciplines indicate that impaired access to care or healthcare status causes delayed hospital presentation among African Americans compared to other races [27–30]. An interesting question is whether white participants may have had better access to quality outpatient infection care than black participants, with only those with more serious illness presenting to the hospital for care. This hypothesis is tempered by the fact that we observed similar SOFA and MEDS scores between races. The contrasting findings between the current and prior studies may have also occurred if black participants were more likely than white participants to develop hospital-acquired sepsis. Mayr, et al. (2010) found that black participants were more likely to develop postoperative infections than their white counterparts, which would support this hypothesis [6]. Because of our focus on community-acquired sepsis, we did not examine the later parts of the hospital course to identify the development of hospital-acquired sepsis.

Racial disparities are important because they may reflect differences in biological response, genetics, health behaviors, or access to or quality of medical care. Studies of hepatitis C virus and coronary heart disease have affirmed the presence of racial disparities due to differences in all of these domains [31–33]. In the national effort to reduce sepsis morbidity and mortality, racial differences might shed light on the pathophysiologic or clinical factors that influence sepsis outcomes. Some may view our results as negating the need for scrutinizing racial disparities. On the contrary, our findings - especially as they contrast with prior studies - signal the need to more intensely explore racial differences.

### Limitations

REGARDS is a longitudinal study intended to investigate stroke, and not sepsis outcomes. The current analysis is not a surveillance study, and thus we may not have achieved complete ascertainment of all sepsis events. Recall bias is a limitation, and therefore the number of sepsis events identified may be underestimates. By design, the REGARDS cohort includes only African Americans and white Americans, and thus these results may not be generalizable to other ethnic groups. REGARDS oversampled black participants in the southeastern US, potentially limiting generalizability to other populations.

Selection bias or participation bias are potential limitations of this study. By design, the REGARDS cohort contains individuals over 45 years old only; sepsis rates and the observed associations may have differed for younger individuals. Thus, it is possible that black participants may have experienced more sepsis-related events at younger ages than white participants, an observation made in previous research [4]. Participants may have been less likely to participate in the baseline visit of study based on work status (i.e., black men were less likely to participate). This is largely explained by required blood management visits at baseline during morning weekdays, which may have alternatively required missed workdays. However, this is likely an equal burden for all race-sex strata, but could have been more prevalent for black men. We identified individuals who presented to the hospital with sepsis, but did not include those who acquired sepsis during their hospitalization. Participants in the REGARDS cohort could be significantly different from prior studied populations. However, previous studies investigating the REGARDS cohort have observed racial differences consistent with other cohorts; for example, black participants having higher risk of fatal coronary heart disease than white participants [32].

REGARDS did not have information on vaccination, so we could not ascertain if racial differences in vaccinations may have resulted in different infection rates. Prior research suggests that black patients have a higher incidence of infections (e.g., bacteremic pneumonia) than white patients [34, 35]. Robinson, et al. explained that the racial disparity could be attributed to lower vaccination rates among black patients [35]. Furthermore, an additional study found that after the introduction of the pneumococcal vaccine, the disparity between black and white participants diminished [36].

## Conclusion

In conclusion, in the national REGARDS cohort, black participants were less likely than white participants to experience infection and sepsis events. However, after hospitalization for first infection there were no racial differences in the odds of sepsis. Further efforts should focus on elucidating the underlying reasons for these observations, which are in contrast to existing literature.

## Key messages

- In the REGARDS cohort, the long-term risk of community-acquired sepsis is higher in white participants than in black participants.
- In this study, white participants were more likely to develop both infection and sepsis.
- Among those with first infections, the odds of sepsis were not different between black and white participants.

## Abbreviations

ACR: albumin-to-creatinine ratio
BMI: body mass index
CKD-EPI: Chronic Kidney Disease Epidemiology Collaboration equation
CRP: C-reactive protein
Cyst-C: Cystatin-C
ECG: electrocardiogram
eGFR: estimated glomerular filtration rate
HR: hazard ratio
MEDS: mortality in emergency department
MI: myocardial infarction
OR: odds ratio
REGARDS: Reasons for geographic and racial differences in stroke
SIRS: systemic inflammatory response syndrome
SOFA: sequential organ failure assessment
WC: waist circumference
